# CpG methylation profiling in *VHL *related and *VHL *unrelated renal cell carcinoma

**DOI:** 10.1186/1476-4598-8-31

**Published:** 2009-06-03

**Authors:** Fiona E McRonald, Mark R Morris, Dean Gentle, Laura Winchester, Dilair Baban, Jiannis Ragoussis, Noel W Clarke, Michael D Brown, Takeshi Kishida, Masahiro Yao, Farida Latif, Eamonn R Maher

**Affiliations:** 1 Cancer Research UK Renal Molecular Oncology Group, University of Birmingham, Birmingham, B15 2TT, UK; 2 Section of Medical and Molecular Genetics, Department of Paediatrics and Child Health, University of Birmingham, Birmingham, B15 2TT, UK; 3 Genomics Laboratory, Wellcome Trust Centre For Human Genetics, University of Oxford, Roosevelt Drive, Oxford, OX3 7BN, UK; 4 The GU Research Group, Christie Hospital and Paterson Institute for Cancer Research, University of Manchester, Manchester, M20 4BX, UK; 5 Department of Urology and Molecular Geneticis, Yokohama City University Graduate School of Medicine, Yokohama, Japan

## Abstract

**Background:**

Renal cell carcinoma (RCC) is histopathologically heterogeneous with clear cell and papillary the most common subtypes. The most frequent molecular abnormality in clear cell RCC is *VHL *inactivation but promoter methylation of tumour suppressor genes is common in both subtypes of RCC. To investigate whether RCC CpG methylation status was influenced by histopathology and *VHL *status we performed high-throughput epigenetic profiling using the Illumina Goldengate Methylation Array in 62 RCC (29 RCC from von Hippel-Lindau (VHL) disease patients, 20 sporadic clear cell RCC with wild type VHL and 13 sporadic papillary RCC).

**Results:**

43 genes were methylated in >20% of primary RCC (range 20–45%) and most (37/43) of these had not been reported previously to be methylated in RCC. The distribution of the number of methylated CpGs in individual tumours differed from the expected Poisson distribution (p < 0.00001; log-likelihood G test) suggesting that a subset of RCC displayed a CpG Island Methylator Phenotype. Comparison of RCC subtypes revealed that, on average, tumour specific CpG methylation was most prevalent in papillary RCC and least in VHL RCC. Many of the genes preferentially methylated in pRCC were linked to TGFβ or ERK/Akt signalling.

**Conclusion:**

These findings demonstrate differing patterns of tumour-specific CpG methylation in VHL and non VHL clear cell RCC and papillary RCC, and identify multiple novel potential CpG methylation biomarkers for RCC.

## Introduction

Renal cell carcinoma (RCC) accounts for 2–3% of all cancers, and most kidney cancers arise from the renal tubule epithelium. The most common types of RCC, accounting for ~90% of tumours, are conventional (clear cell) renal cell carcinoma (cRCC) and papillary (pRCC). Investigations of rare inherited forms of RCC have provided insights into the molecular pathogenesis of both familial and sporadic RCC. Thus the identification of the gene for von Hippel-Lindau (VHL) disease (a dominantly inherited familial cancer syndrome characterised by the development of retinal and central nervous system haemangioblastomas, cRCC, pancreatic lesions and phaeochromocytoma) led to the recognition that the most frequent genetic event in the evolution of sporadic cRCC is somatic inactivation of the *VHL *tumour suppressor gene (TSG) [1-4]. However, *VHL *inactivation is not a feature of pRCC.

Epigenetic inactivation of TSGs by methylation of promoter region CpG dinucleotides has been frequently implicated in the pathogenesis of human cancers including RCC. Thus epigenetic silencing of VHL may occur in up to 20% of sporadic cRCC [4-6]. Although VHL promoter methylation is not a feature of pRCC, methylation of some TSGs, e.g. *RASSF1A *and *SPINT2*, occurs in both cRCC and pRCC [7,8]. Relatively little is known about how pathways of tumourigenesis in cRCC with and without *VHL *inactivation compare, and, specifically, whether epigenetic changes differ according to whether VHL is inactivated or not. Epigenetic profiling to detect TSG promoter methylation is an efficient strategy for investigating tumourigenesis pathways in RCC. In contrast, with the exception of *VHL*, the frequency of mutations in individual candidate TSGs in RCC is <15% , however, we and others have identified at least 14 candidate TSGs demonstrating tumour-specific promoter methylation in >20% of RCC ([8,9], and references within). Furthermore, recent technological developments have enabled analysis of CpG methylation to be undertaken for many TSGs simultaneously. In order to (a) gain a better understanding of the frequency and nature of TSG methylation in RCC and (b) compare the patterns of CpG methylation in TSGs from papillary RCC and cRCC with and without VHL inactivation, we analysed RCC samples using a high-throughput CpG methylation analysis platform (Illumina Goldengate Assay).

## Methods

### Tumour samples

Genomic DNA was extracted from primary renal cancers and cell lines by standard methods, and stored at -80°C. Three groups of renal cancers were investigated: (a) 29 cRCC from patients with von Hippel-Lindau disease, (b) 20 sporadic cRCC without evidence of somatic VHL mutations or promoter methylation (details of mutation and methylation analyses have been reported previously [4]) and (c) 13 papillary RCC. In addition, DNA samples from normal kidney tissue (NKT) from patients without cancer (n = 6, mean age 57 years, range from 23–79 years) and 24 kidney cancer cell lines were studied (786-0, 769P, A498, A704, ACHN, Caki1, Caki2, CAL54, KTCL26, KTCL140, NK2, RCC1, RCC4, RCC6, RCC11, RCC12, RCC48, SKRC18, SKRC39, SKRC45, SKRC47, SKRC54, UMRC2 and UMRC3). Ethical approval for collection of clinical material was obtained from the South Birmingham Ethics Committee and relevant local ethics committees.

### Methylation Studies

#### Illumina Goldengate Methylation Analysis

0.5 μg DNA samples were treated with sodium bisulphite using the EZ DNA methylation Gold kit (Zymo), and the bisulphite-treated DNA was applied to an Illumina bead array [10] using the Illumina Goldengate Methylation Cancer Panel  (performed at the Wellcome Trust Centre for Human Genetics, University of Oxford). Methylation results were analysed in a qualitative fashion (akin to conventional methylation analysis using Methylation Specific PCR (MSP)), such that a tumour was considered to be positive for CpG methylation if the array detected >25% methylation (this would correspond to complete monoallelic CpG methylation in a tumour that contained 50% contaminating normal tissue). 84 genes were methylated (Mt > 0.25 in all 6 NKT but unmethylated (MtI ≤ 0.25) in one or more primary tumours. To allow for normal variation, attention was focussed on 18 genes that were methylated in 100% of NKT but <72% (lower 95% CI) of primary renal tumours tested.

#### Validation of Illumina Goldengate methylation data

Prior to the current experiments, reproducibility was established during alternative experiments using replicates of several sample types including Universally Methylated Controls. These demonstrated high correlations between repeated samples (r^2 ^= 0.93–0.85). Three genes with a total of four CpGs (EYA4_P508_F, EYA4_P794_F, SOX17_P287_R, TNFRSF10C_E109_F) were analysed by direct bisulphite sequencing in 6 RCC cell lines (RCC48, CAL54, CAKI2, RCC1, RCC11, RCC12, 786P). 23 of 24 CpGs showed evidence of methylation by direct sequencing, and 22 of these were categorised as methylated by the array analysis. One tumour with partial methylation by direct sequencing at EYA4_P508_F was below the methylation threshold on the array analysis (methylation index, MtI = 0.15). The remaining CpG, EYA4_P508_F in RCC cell line 769-P, was unmethylated by both direct sequencing and array analysis. Overall, therefore, there was close correlation between the methylation results obtained by the two methods and no evidence of a significant difference (McNemar test P = 1.0). In addition, inspection of the broader sequence context of the particular CpGs targeted by the Illumina array showed that the methylation status of these individual CpGs was representative of the methylation status of the surrounding CpGs.

### Statistical and Bioinformatic Analysis

Kruskal-Wallis Test, log-likelihood G test, log-likelihood G test for Goodness of Fit against Poisson Distribution, and Pearson product-moment correlation coefficient analyses were performed as appropriate. As the aim of the research was merely to catalogue a list of plausible candidate tumour suppressor genes for RCC, without seeking to prove their involvement or causality, the use of a Bonferroni correction would have been unnecessarily restrictive, especially given the large number of CpGs interrogated by the Goldengate assay (n = 1505). P-values are therefore given for indicative purposes only, with the acknowledgement that a rigorous correction for multiple statistical testing has not been applied.

Hierarchical cluster analysis was performed using Illumina BeadStudio software  with the Euclidean algorithm (identical results were obtained with the Manhattan algorithm) (see ) and used to create a heatmap with associated dendrogram. Functional characteristics of genes of interest were examined using Ingenuity Systems software .

## Results

### Epigenetic Profile of Human Renal Cancers and Cell Lines

**Figure 1:**
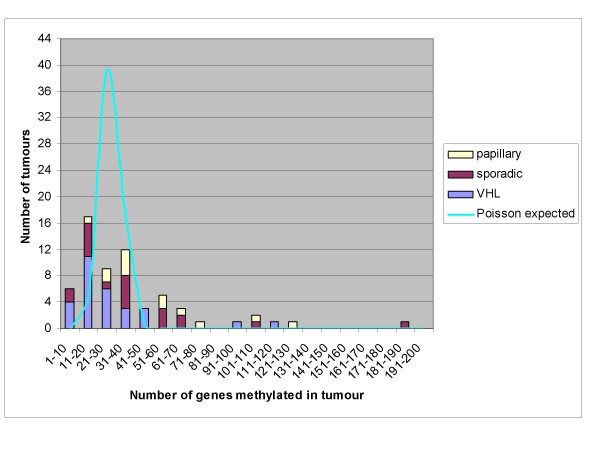
**Distribution of the number of genes found to be >25% methylated in each tumour, according to histological type**. Poisson probabilities are based on the geometric mean of the total observed data (i.e. λ = 27.609) Geometric mean number of genes methylated for each tumour type is indicated by coloured circles on the x-axis.

**Figure 2:**
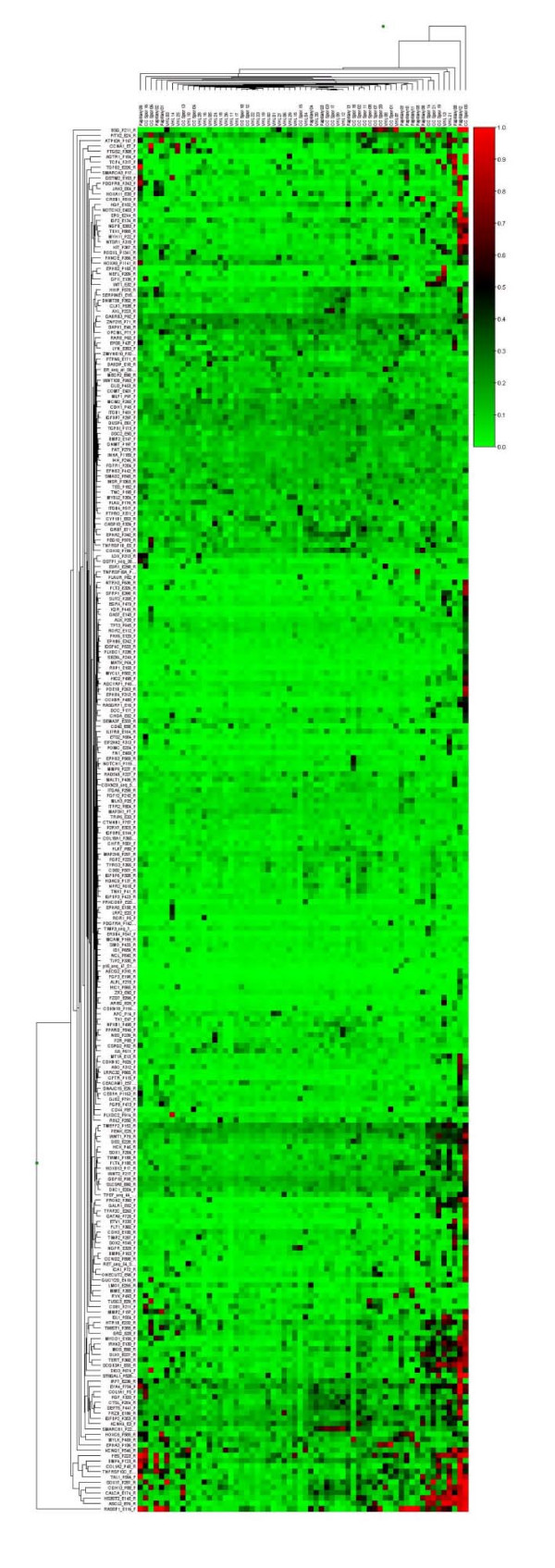
**Heat map showing gene (n = 260) methylation patterns in 62 primary RCC**. Euclidean cluster analysis demonstrates preferential grouping of VHL tumours (VHL 01 – 29) versus non-VHL sporadic cRCC (CC Spor 01 – 21) and papillary tumours (papillary 01 – 13). Tumours are shown on the x-axis. CpG sites from 260 candidate genes are shown on the y-axis. Methylation is colour coded from 0% (green) to 100% (red)

The Illumina GoldenGate Methylation Cancer Panel I array provides quantitative CpG methylation data at 1505 individual CpG dinucleotides associated with 807 human genes. In order to profile patterns of RCC-specific candidate TSG methylation, we initially excluded 547 genes from further analysis because (a) they were methylated in Normal Kidney Tissue (NKT) (n = 338, this included X chromosome genes), (b) were methylated only in cell lines (n = 42), (c) were unmethylated in all samples studied (n = 101) or (d) a further 66 that fell into more than one of the above three categories (genes could be in >1 category as most genes had >1 CpG interrogated). 260 genes were methylated in tumour but not in NKT, and Euclidean cluster analysis was performed on these genes (see later). The number of methylated genes per individual tumour ranged between 4 and 117. The distribution of the number of methylated CpGs in individual tumours differed from the expected Poisson distribution (p < 0.00001; log-likelihood G test) (see figure 1). This suggested that the extent of methylation in individual tumours was not randomly distributed, and that a subset of RCC tumours might display CpG Island Methylator Phenotype (CIMP+ tumours) (see Figures 1 and 2).

43 of 260 candidate non-imprinted tumour suppressor genes were methylated in ≥ 20% (range 20–45%) of tumours (these were designated "frequently methylated" genes). These genes were analysed in detail, and are listed in Additional file 1. In most cases (37/43 genes), methylation was more frequent in cell lines than primary tumours, however for 6 genes this pattern was reversed (*ZNF215*: 21% and 42% respectively, *DAPK1*: 4% and 39%, *EPHA3 *13% and 32%, *SMARCB1*: 8% and 27%, *EPS8 *4% and 21% and *MCM2: *0% and 21%).

**Figure 3:**
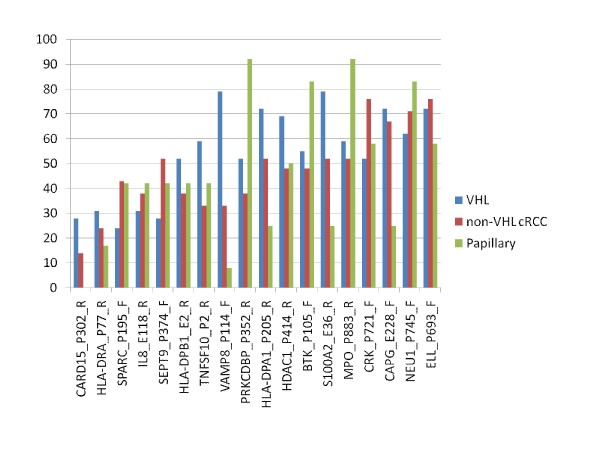
**Genes that were methylated in all normal kidney tumour samples (N = 6) but methylated in <72% of renal tumours analysed: comparison of frequency of methylation in different tumour types**.

18 genes were methylated in all six normal kidney tissue samples but in <72% of renal tumours analysed: CARD15 (18% of tumours), HLA-DRA (24%), SPARC (34%), IL8 (35%), SEPT9 (39%), HLA-DPB1 (45%), TNFSF10 (47%), VAMP8 (50%), PRKCDBP (55%), HLA-DPA1 (56%), HDAC1 (58%), BTK (58%), S100A2 (60%), MPO (61%), CRK (61%), CAPG (61%), NEU1 (69%), ELL (71%). There was no consistent pattern in the frequency of methylation loss in the different subtypes of renal tumours (Figure 3).

### Analysis of Methylation Patterns according to RCC Subtype

The overall distribution of methylation between the three groups of tumours (VHL cRCC, sporadic wtVHL cRCC and pRCC) was significantly different (p < 0.006; Kruskal-Wallis Test). Thus, papillary RCCs demonstrated a geometric mean of 62.5 tumour-specific methylated CpGs per tumour, sporadic cRCCs had a geometric mean of 25.5 and VHL-associated cRCCs 20.9 methylated CpGs per tumour (See Figure 1). Nevertheless, 15 genes (*EYA4, KCNK4, ZNF215, DAPK1, TMEFF2, SEPT5, SOX17, PENK, CTSL, BMP4, HTR1B, COL1A2, FRZB, SMARCB1*, and *CCNA1*) were each methylated in >20% of the three subtypes and did not show significant differences in methylation frequency between tumour subtypes. Comparison of CpG methylation patterns in cRCC (VHL and sporadic wtVHL) and pRCC demonstrated that (at a 1% significance level using log-likelihood G-test) one gene (*CDH1*) was more methylated in the cRCC (29% vs 0%; p < 0.01) and 14 genes were more methylated in the papillary RCC (*RASSF1*, *SERPINE1*, *HOXA11*, *HOXC6*, *JAK3, PDGFRB, MMP2, ITGB1, CREB1, MYOD1, GSTM2, TNFRSF10C, SMARCA3 *and *COL1A1 *(see Additional file 2). We considered that differences in methylation profiles between different tumour types might be related to differing mechanisms of tumourigenesis or, potentially, to ascertainment bias as most VHL tumours are detected presymptomatically (via renal imaging surveillance programmes) and so will tend to be smaller than sporadic tumours. Indeed mean (±SD) tumour diameter in VHL patients was 2.46 ± 0.56 compared to 6.3 ± 4.0 cm in wtVHL-cRCC and 5.0 ± 3.6 in papillary RCC. However, although, on average, pRCC were smaller than wtVHL-cRCC, tumour-specific CpG methylation was generally higher in pRCC (see above) and 12 genes were more frequently methylated in pRCC than in wtVHL-cRCC (*SERPINE1, HOXC6, HOXA11, JAK3, ITGB1, PDGFRB, RARB, GSTM2, MYOD1, SMARCA3*(*HTLF*), *GABRB3 *and *TGFB2*), whereas only 5 genes were more frequently methylated in wtVHL-cRCC than pRCC (*TWIST1, TIAM1, DCC, CDH1 *and *PTGS2*) (all at p ≤ 0.025 (see Additional file 2)), There was a significantly positive correlation (Pearson product-moment correlation coefficient; p < 0.05) between tumour size and gene methylation in only three genes with differential methylation between tumour types (*PDGFRB, TIAM1 *and *GSTM2*), suggesting that in most cases the observed differential methylation is related to different tumour histologies rather than to tumour size.

Comparison of methylation patterns in VHL cRCC and sporadic wtVHL cRCC demonstrated that 11 frequently methylated genes (i.e. genes methylated in > 20% of at least one tumour type) were significantly (p < 0.025) more frequently methylated in wtVHL sporadic RCC than in VHL RCC (*RASSF1, TWIST1 PITX2*, *CDH13, HS3ST2*, *TAL1, WT1*, *MMP2*, *DCC*, *ICA1 *and *TUSC3*:(all p < 0.025; log-likelihood G-test; see Additional file 2). One frequently methylated gene was significantly more frequently methylated in VHL-associated cRCC than in wtVHL sporadic cRCC: *GABRB3 *(48% vs 20%; log-likelihood G-test, p < 0.05; a less stringent p-value was used due to the lower overall frequency of methylation in the VHL subgroup). For genes more methylated in wtVHL than VHL-RCC, *RASSF1, PITX2, CDH13, HS3ST2, TAL1, TUSC3 *and *DCC *were at least as frequently methylated in tumours < 5 cm as those ≥ 5 cm. Euclidean cluster analysis for 260 genes showing tumour-specific methylation was performed (this analysis used quantitative methylation index data) and there was significant evidence of clustering of tumour types (comparing VHL and non-VHL tumours (Fisher exact P = 0.00042) and using all three types χ^2 ^= 13.3, df = 2, P = 0.0013) (see Figure 2)

**Figure 4:**
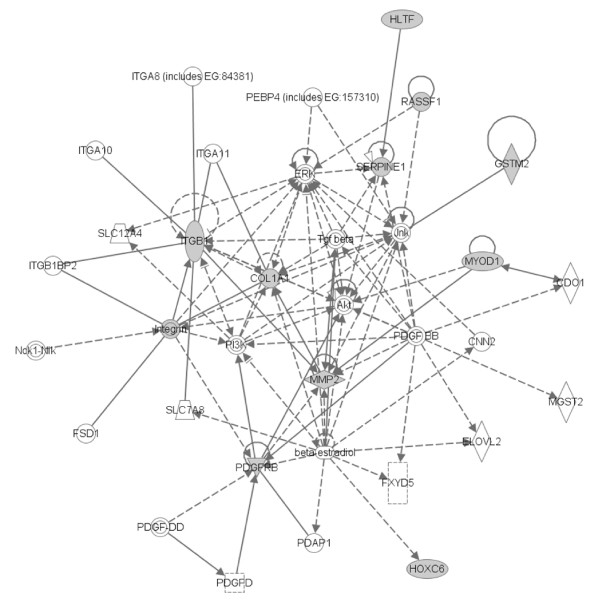
**Most significant gene networks for genes more methylated in papillary RCC than clear cell RCC**. Genes in unfilled nodes were not identified as specifically methylated in tumours (TGF-β, AKT, PDGFB) or were not represented on the array (ITGA8, ITGA10, ITGA11, PEBP4, ERK, SLC12A4, JNK, ITGB1BP2, CDO1, NCK1, NLK, PI3K, CNN2, FSD1, SLC7A8, ELOVL2, FXYD5, PDGFD and PDAP1. The computationally generated networks were derived using the Ingenuity package . (see Figure 6 for key to nodal shape).

**Figure 5:**
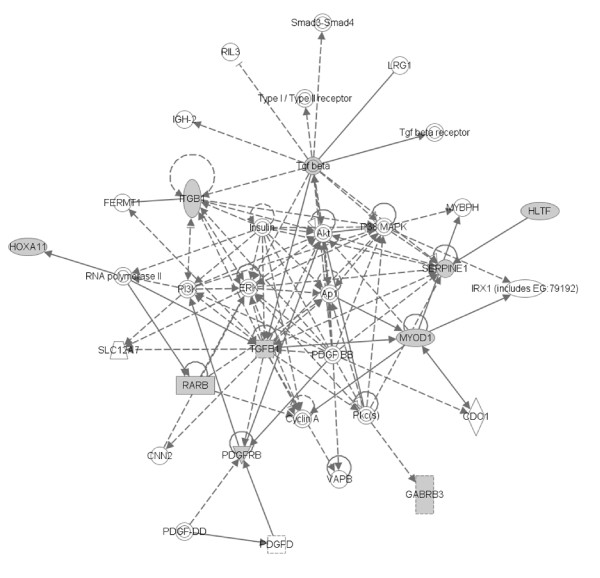
**Most significant gene networks for genes more methylated in papillary RCC than non-VHL sporadic cRCC**. Genes in unfilled nodes were not identified as specifically methylated in tumours (AKT), were not represented on the array (SMAD3/SMAD4, RIL3, LRG1, TGFBR, FERMT1, P38MAPK, MYBPH, RNA polymerase II, insulin, PI3K, ERK, Ap1, SLC12A7, PDGFB, Pkc(s), CDO1, CNN2, VAPB, PDGFD) or were methylated in tumours but with no frequency difference between tumour types (CCNA1). The computationally generated networks were derived using the Ingenuity package  (see Figure 6 for key to nodal shape).

**Figure 6:**
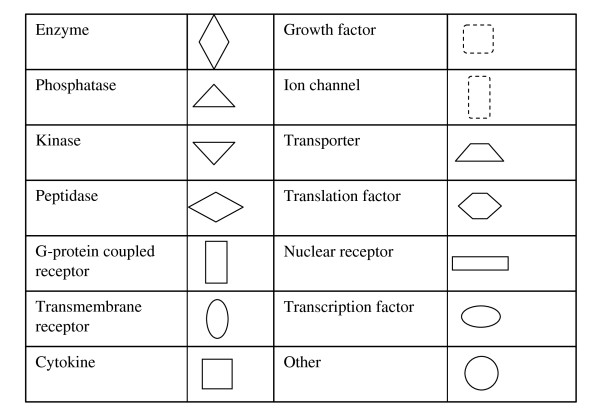
**Key to nodal shape in figures 4 and 5**.

To determine if differential CpG methylation patterns in RCC might relate to preferential targeting of specific signalling pathways, the Ingenuity functional annotation pathway was utilised. 8/14 genes that were significantly more methylated in pRCC than cRCC were represented in a network with links to TGFβ and ERK/Akt pathways (see Figure 4). The link to these pathways was more pronounced (9/12) for those 12 genes that were more frequently methylated in pRCC than in wtVHL-cRCC (see Figure 5 (see figure 6 for key to nodal shape in figures 4 and 5). The 18 genes that were methylated in all NKT samples but <72% of renal tumours did not demonstrate a significant association with a particular pathway.

## Discussion

High throughput methylation profiling platforms such as the Illumina Goldengate assay enable extensive methylation profiling of human tumours for a large number of genes. Although many of the genes represented in the assay have previously been reported as methylated in human cancers, most (33/42) of the candidate tumour suppressor genes that were methylated in ≥ 20% of all primary RCC tested had not previously been reported to be methylated in RCC (*HTR1B, CALCA, IGFBP2, SOX17, COL1A2, BMP4, HS3ST2, FRZB*, *TAL1, MCM2, KCNK4, HOXC6, PITX2, SEPT5, IRF7, CCNA1, HOXA11, TERT, TMEFF2, EPHA3, PGF, MYOD1, MMP2, TNFRSF10C, PENK, EYA4, MYLK, IRAK3, ZNF215, SMARCB1, TWIST1, SCGB3A1*, and *IGFBP7*). Thus our analysis suggests a large number of potential epigenetic biomarkers and/or TSGs for RCC. In addition, several of these genes have not previously been reported as methylated in any type of cancer (*KCNK4, SEPT5, PENK, BMP4, TAL1, PGF, SMARCB1 (INI1), FRZB (SFRP3), IRAK3 *and *MCM2*). Detection of methylated CpGs in urine has been investigated as a potential screening tool for RCC and other urinary tract neoplasms (reviewed in ref 11). To date, no single gene is known to be hypermethylated in all RCCs, and so successful application of promoter methylation assays as biomarkers will necessitate the study of multiple loci. Ideally the combination of loci chosen for such a screening strategy would cover all types of RCC. Such a panel might include genes that are (a) methylated in all types of RCC and (b) preferentially methylated in a particular subtype of RCC, as such a combination could provide a basis for minimally-invasive screening for diagnosis, prognostication and therapeutic targeting. The potential for such a strategy is demonstrated by the results of Euclidean cluster analysis that grouped most tumours into groups consistent with their histopathology/VHL status.

We found evidence that a small number of RCC harboured more methylated CpGs than would be expected by chance. This provides evidence for a "CpG island methylator phenotype" (CIMP+) in a subset of RCC. CIMP+ tumours were not specific to any subgroup of RCC. The concept of a CIMP+ phenotype was developed from studies of colorectal cancer and remained controversial until relatively recently. However, using an unbiased approach to defining putative CIMP-related markers, Weisenberger et al (a) provided evidence for CIMP+ colorectal cancer and (b) demonstrated that CIMP+ sporadic colorectal cancers were associated with the presence of a *BRAF *mutation and microsatellite instability (from *MLH1 *promoter methylation) (12). Although CIMP has been described in a range of other tumour types (including head and neck squamous cell carcinoma, bladder cancer, non-small cell lung cancer and malignant pleural mesothelioma) [13], evidence for a CIMP in RCC has not been reported previously. However, we note that Dulaimi et al [14] reported that a subset of RCC (~3%) were methylated for at least five out of the ten genes studied. Identification of further CIMP+ RCCs will provide a basis for determining whether tumours with extensive CpG methylation have distinct clinical characteristics and whether a subset of genes are methylated only in CIMP+ phenotype tumours. The molecular causes of CIMP are not well-understood and identification of CIMP+ tumours will facilitate further studies to investigate the molecular basis of this phenotype [15].

We were interested to compare the methylation profiles of cRCC and papillary RCC, as little information is available on this topic. Several genes were significantly (p < 0.025) more frequently methylated in papillary than in cRCC (and similarly methylated in both cRCC subtypes). These loci could prove to be useful candidate biomarkers for papillary RCC and might give insights into differing mechanisms of tumourigenesis in papillary and cRCC, thereby enable focusing of targeted therapeutic drugs in different disease sub-types. Recently, Matsuda et al [16] undertook high resolution array CGH studies in sporadic RCC and reported that whereas cRCC was characterized by frequent 3p loss and 5q gain, papillary RCC demonstrated frequent gains on chromosomes 2, 7, and 12; additionally, loss on 1q, 9, and 11q was unique to papillary RCC. However none of the loci preferentially methylated in papillary RCC mapped to 1q, 9 or 11q; and *RARB*, although preferentially methylated in papillary RCC, maps to 3p24. Thus loci with high frequencies of CpG methylation may occur in regions with a low frequency of allele loss. It is noteworthy that both *HOXA11 *and *HOXC6 *were preferentially methylated in papillary RCC. However, although evidence for preferential methylation of genes within a specific chromosome region has been reported in colon cancer [17], *HOXA11 *and *HOXC6 *map to 7p15-p14 and 12q13.3 respectively and so their preferential involvement in papillary RCC presumably reflects their related functions rather than their cytogenetic location. Three genes, *CDH1*, *PTGS2 *and *TWIST1 *were specifically methylated in cRCC (both p < 0.025 compared to papillary RCC). Germline mutations in *CDH1 *and *TWIST1 *may be associated with inherited cancer susceptibility. Thus, *CDH1 *mutations cause familial diffuse type gastric cancer [18] and the *TWIST1 *transcription factor is mutated in Saethre-Chotzen syndrome, which is characterised by developmental defects (craniosynostosis and digital anomalies) and is also reported to be associated with an increased risk of breast cancer [19]. *PTGS2 *(*COX2*) encodes prostaglandin-endoperoxide synthase 2, and recently Costa et al [20] also reported that *PTGS2 *methylation levels were significantly higher in cRCC than papillary RCC. Thus these three genes might prove to be useful epigenetic biomarkers for the diagnosis of cRCC (e.g. in needle aspirates). We note that many of the genes that were preferentially methylated in pRCC could be linked in to TGF-β/ERK/Akt pathways. Although the role of TGF-β-related pathways have not been investigated in pRCC, we note that TFE3 may regulate TGF-β signalling [21] and that a subgroup of pRCC are charcaterised by chromosomal translocations involving TFE3 [22].

Although most sporadic cRCC have evidence of *VHL *inactivation, little is known about whether *VHL*-inactivated and *VHL*-wt RCC have differing or similar (for *VHL*-independent events) mechanisms of tumourigenesis. Also, there is little information on the somatic changes (other than *VHL *inactivation) associated with RCC from VHL patients. To our knowledge, this is the first investigation of epigenetic alterations in *VHL*-null and *VHL*-wt RCC. We found that a number of loci, including *RASSF1*, *PITX2, CDH13*, *HS3ST2*, *TWIST1, TAL1*, *TUSC3 *and *DCC *were significantly more frequently methylated in VHL-wt sporadic RCC than in VHL RCC. It might be hypothesised that genes whose functions overlap with that of *VHL*-related pathways might be preferentially inactivated in RCC without *VHL *inactivation. Therefore it is interesting that both VHL and RASSF1A downregulate Cyclin D1 [23-25]. However, although *CDH1 *expression is downregulated by VHL inactivation, and *DAPK1 *by *RASSF1A *inactivation, *CDH1 *and *DAPK1 *methylation frequencies were similar in wtVHL sporadic RCC and VHL RCC. Also we note that all of the genes that were preferentially methylated in wtVHL sporadic RCC mapped to different chromosomal locations, and there was no evidence that a specific chromosomal domain was more frequently methylated in a specific tumour type. 5 genes that were more frequently methylated in sporadic wtVHL-cRCC than VHL RCC were included in a network that related to ERK/Akt signalling. Both the Raf-extracellular signal-regulated protein kinase (Erk)1/2 and Akt-mTOR pathways have been implicated in the pathogenesis of familial and sporadic RCC [26,27]. *VHL*-inactivation may be associated with activation of the epidermal growth factor receptor/phosphatidylinositol-3-OH kinase/protein kinase B (AKT)/IkappaB-kinase alpha/NF-kappaB signalling cascade [28], and so RCC without *VHL *inactivation may be predisposed to dysregulate these key signalling pathways by preferential methylation of other regulators. Although it might be predicted that the pathways of tumourigenesis in VHL mutated sporadic clear cell RCC will be similar to those in RCC from VHL patients, further studies (using the methods utilised in this study) are indicated to investigate this point.

It is generally thought that CpG island methylation in cancer is non-random, and many genes that are frequently methylated have been shown to have a tumour suppressor or DNA repair function. TSGs may be targeted by epigenetic silencing and/or inactivating mutations during tumourigenesis. However, whereas the repertoire of inactivating mutations in a typical tumour suppressor gene is extensive, patterns of CpG island methylation causing epigenetic silencing are much more restricted and hence are easier to detect. The relative frequency of CpG island methylation and inactivation mutations for individual TSGs is variable. For example, although *VHL *is primarily inactivated by somatic mutations (promoter methylation occurs in ~15% of sporadic cRCC), inactivation of the *RASSF1A *TSG in RCC (and in other tumour types) most commonly results from promoter methylation, whilst intragenic mutations are rare. According to the Catalogue of Somatic Mutations in Cancer (COSMIC; ) only three genes are mutated in >5% of RCC tested (VHL = 42%, CDKN2A = 12% and KIT = 8%). Thus, with the exception of *VHL*, epigenetic biomarkers are likely to provide a better approach for novel prognostic and diagnostic strategies than genetic markers [29]. This is substantiated by the observation that, of the 43 genes that were methylated in >20% of RCC tested, RCC mutation analysis data is available for 21, of which none are subject to somatic mutations in RCC. Further characterisation of the genes and pathways that are epigenetically altered in RCC of different subtypes may thus lead to the development of novel minimally-invasive diagnostic and prognostic tools for kidney cancer, and, in the longer term, may enable more focused treatments for individual tumours.

## Competing interests

The authors declare that they have no competing interests.

## Authors' contributions

FMcR participated in the design of the study carried out the molecular genetic studies, analysed data, performed statistical analysis and co-wrote the paper. MRM and DG carried out the molecular genetic studies. LW carried out the molecular genetic studies, analysed data and performed statistical analysis. DB and IR performed bioinformatic analysis. NWC, MDB, TK and MY provided tissue and tumour samples. FL participated in the design of the study. and performed the statistical analysis. ERM conceived the study, participated in the design and coordination of the study, analysed data, performed statistical analysis and co-wrote the paper. All authors read and approved the final manuscript.

## Supplementary Material

Additional file 1 **Details of genes methylated in >20% of all RCC tested**. A table containing details of 43 candidate non-imprinted tumour genes that were methylated in ≥ 20% of tumours. Click here for file

Additional file 2 **Comparison of gene methylation frequency between tumours of different histological subtypes**. Data and statistical analysis of gene methylation in different RCC subtypes. Click here for file
