# Supplementary material for: CpG methylation profiling in VHL related and VHL unrelated renal cell carcinoma
Source: Mol Cancer. 2009 Jun 3;8:31. doi: 10.1186/1476-4598-8-31 (PMC2698845; doi:10.1186/1476-4598-8-31)
Supplement: Additional file 1 — Details of genes methylated in >20% of all RCC tested. A table containing details of 43 candidate non-imprinted tumour genes that were methylated in ≥ 20% of tumours. [file 1476-4598-8-31-S1.doc]

**Additional File 1: Details of genes methylated in >20% of all RCC tested.**

| **Gene** | **Illumina Target ID** | **% of methylated tumours** | | | | | | | **Reference if reported to be methylated in RCC** |
| --- | --- | --- | --- | --- | --- | --- | --- | --- | --- |
|  |  | **All RCC** | **cRCC (VHL & sporadic)** | **VHL RCC** | | **Sporadic cRCC** | **Papillary RCC** | **RCC cell lines** |  |
| EYA4 | EYA4_P794_F | 45.2 | 40.8 | 34.5 | 50 | | 61.5 | 87.5 |  |
| RASSF1 | RASSF1_E116_F | 43.5 | 32.7 | 13.8 | 60 | | 84.6 | 95.8 | 20 |
| KCNK4 | KCNK4_E3_F | 43.5 | 42.9 | 48.3 | 35 | | 46.2 | 100.0 |  |
| GABRB3 | GABRB3_P92_F | 41.9 | 36.7 | 48.3 | 20 | | 61.5 | 58.3 |  |
| ZNF215 | ZNF215_P71_R | 41.9 | 36.7 | 37.9 | 35 | | 61.5 | 20.8 |  |
| PITX2 | PITX2_E24_R | 41.9 | 38.8 | 24.1 | 60 | | 53.8 | 66.7 |  |
| DAPK1 | DAPK1_E46_R | 38.7 | 42.9 | 37.9 | 50 | | 23.1 | 4.2 | 29 |
| TNFRSF10C | TNFRSF10C_E109_F | 37.1 | 28.6 | 24.1 | 35 | | 69.2 | 95.8 |  |
| MYLK | MYLK_P469_R | 37.1 | 30.6 | 27.6 | 35 | | 61.5 | 83.3 |  |
| TMEFF2 | TMEFF2_P152_R | 37.1 | 32.7 | 27.6 | 40 | | 53.8 | 45.8 |  |
| SEPT5 | SEPT5_P441_F | 37.1 | 32.7 | 27.6 | 40 | | 53.8 | 83.3 |  |
| SOX17 | SOX17_P287_R | 37.1 | 36.7 | 27.6 | 50 | | 38.5 | 95.8 |  |
| PENK | PENK_E26_F | 35.5 | 36.7 | 27.6 | 50 | | 30.8 | 87.5 |  |
| CDH13 | CDH13_P88_F | 33.9 | 28.6 | 13.8 | 50 | | 53.8 | 91.7 |  |
| CTSL | CTSL_P264_R | 33.9 | 30.6 | 27.6 | 35 | | 46.2 | 91.7 |  |
| HS3ST2 | HS3ST2_E145_R | 33.9 | 32.7 | 17.2 | 55 | | 38.5 | 95.8 |  |
| TAL1 | TAL1_P594_F | 32.3 | 26.5 | 13.8 | 45 | | 53.8 | 95.8 |  |
| CALCA | CALCA_E174_R | 32.3 | 26.5 | 17.2 | 40 | | 53.8 | 95.8 |  |
| BMP4 | BMP4_P123_R | 32.3 | 26.5 | 27.6 | 25 | | 53.8 | 87.5 |  |
| EPHA3 | EPHA3_P106_R | 32.3 | 36.7 | 34.5 | 40 | | 15.4 | 12.5 |  |
| IGFBP2 | IGFBP2_P353_R | 30.6 | 24.5 | 24.1 | 25 | | 53.8 | 91.7 |  |
| HOXC6 | HOXC6_P585_R | 29.0 | 16.3 | 13.8 | 20 | | 76.9 | 29.2 |  |
| COL1A1 | COL1A1_P5_F | 29.0 | 20.4 | 10.3 | 35 | | 61.5 | 95.8 | 30 |
| PGF | PGF_P320_F | 29.0 | 22.4 | 24.1 | 20 | | 53.8 | 66.7 |  |
| HTR1B | HTR1B_E232_R | 29.0 | 30.6 | 24.1 | 40 | | 23.1 | 79.2 |  |
| COL1A2 | COL1A2_P48_R | 29.0 | 28.6 | 27.6 | 30 | | 30.8 | 95.8 |  |
| FRZB | FRZB_E186_R | 27.4 | 22.4 | 20.7 | 25 | | 46.2 | 91.7 |  |
| SMARCB1 | SMARCB1_P220_R | 27.4 | 26.5 | 24.1 | 30 | | 30.8 | 8.3 |  |
| IGFBP7 | IGFBP7_P297_F | 25.8 | 20.4 | 24.1 | 15 | | 46.2 | 20.8 |  |
| IRF7 | IRF7_E236_R | 25.8 | 22.4 | 27.6 | 15 | | 38.5 | 25.0 |  |
| HOXA11 | HOXA11_E35_F | 24.2 | 14.3 | 10.3 | 20 | | 61.5 | 83.3 |  |
| CCNA1 | CCNA1_E7_F | 24.2 | 22.4 | 20.7 | 25 | | 30.8 | 45.8 |  |
| CDH1 | CDH1_P45_F | 22.6 | 28.6 | 27.6 | 30 | | 0.0 | 45.8 | 20 |
| TERT | TERT_P360_R | 22.6 | 16.3 | 10.3 | 25 | | 46.2 | 87.5 |  |
| SCGB3A1 | SCGB3A1_E55_R | 22.6 | 20.4 | 10.3 | 35 | | 30.8 | 83.3 |  |
| TWIST1 | TWIST1_P355_R | 22.6 | 24.5 | 20.7 | 30 | | 15.4 | 91.7 |  |
| MMP2 | MMP2_P197_F | 21.0 | 12.2 | 3.4 | 25 | | 53.8 | 87.5 |  |
| MYOD1 | MYOD1_E156_F | 21.0 | 12.2 | 10.3 | 15 | | 53.8 | 95.8 |  |
| EPS8 | EPS8_P437_F | 21.0 | 16.3 | 13.8 | 20 | | 38.5 | 4.2 |  |
| CYP1B1 | CYP1B1_E83_R | 21.0 | 24.5 | 20.7 | 30 | | 7.7 | 50.0 |  |
| MCM2 | MCM2_P260_F | 21.0 | 22.4 | 24.1 | 20 | | 15.4 | 0.0 |  |
| IRAK3 | IRAK3_E130_F | 21.0 | 20.4 | 10.3 | 35 | | 23.1 | 91.7 |  |
| HOXA9 | HOXA9_P1141_R | 21.0 | 20.4 | 24.1 | 15 | | 23.1 | 58.3 |  |
