# Supplementary material for: CpG methylation profiling in VHL related and VHL unrelated renal cell carcinoma
Source: Mol Cancer. 2009 Jun 3;8:31. doi: 10.1186/1476-4598-8-31 (PMC2698845; doi:10.1186/1476-4598-8-31)
Supplement: Additional file 2 — Comparison of gene methylation frequency between tumours of different histological subtypes. Data and statistical analysis of gene methylation in different RCC subtypes. [file 1476-4598-8-31-S2.doc]

**Additional file 2: Comparison of gene methylation frequency between tumours of different histological subtypes.**

| **Positive G values for differential methylation between tumour types** | | | | | | | | | | | |
| --- | --- | --- | --- | --- | --- | --- | --- | --- | --- | --- | --- |
| **TargetID** | **SCORE** | **% VHL ccRCC**  **(n=29)** | **% sporadic**  **ccRCC (n=20)** | **% all ccRCC**  **(n=49)** | **% papillary**  **(n=13)** | **% cell lines** | **all**  **G-value** | **clear**  **G-value** | **spor**  **G-value** | **pap v cc G-value** | **Cyto_Map** |
| RASSF1_E116_F | 27 | 13.79 | 60.00 | 32.65 | 84.62 | 95.83 | 23.56^ϒϒ^ | 11.72^ϒϒ^ | 2.40 | 11.85^ϒϒ^ | 3p21.3 |
| SERPINE1_E189_R | 11 | 3.45 | 10.00 | 6.12 | 61.54 | 37.50 | 18.94^ϒϒ^ | 0.87 | 10.16^**^ | 18.07^ϒϒ^ | 7q21.3-q22 |
| HOXC6_P585_R | 18 | 13.79 | 20.00 | 16.33 | 76.92 | 29.17 | 17.37^ϒϒ^ | 0.33 | 10.93^ϒϒ^ | 17.04^ϒϒ^ | 12q13.3 |
| TWIST1_E117_R | 11 | 6.90 | 45.00 | 22.45 | 0.00 | 75.00 | 15.88^ϒϒ^ | 10.11^**^ | 11.15^ϒϒ^ | 5.78* | 7p21.2 |
| MMP2_P197_F | 13 | 3.45 | 25.00 | 12.24 | 53.85 | 87.50 | 14.54^ϒϒ^ | 5.24* | 2.82 | 9.30^**^ | 16q13-q21 |
| HOXA11_P92_R | 6 | 0.00 | 5.00 | 2.04 | 38.46 | 58.33 | 14.16^ϒϒ^ | 1.82 | 6.03* | 12.34^ϒϒ^ | 7p15-p14 |
| JAK3_E64_F | 4 | 0.00 | 0.00 | 0.00 | 30.77 | 87.50 | 13.61^**^ | 0.00 | 8.33^**^ | 13.61^ϒϒ^ | 19p13.1 |
| COL1A1_P5_F | 18 | 10.34 | 35.00 | 20.41 | 61.54 | 95.83 | 12.19^**^ | 4.40^#^ | 2.25 | 7.79^ϒ^ | 17q21.33 |
| HOXA11_E35_F | 15 | 10.34 | 20.00 | 14.29 | 61.54 | 83.33 | 11.98^**^ | 0.88 | 5.92* | 11.09^ϒϒ^ | 7p15-p14 |
| ITGB1_P451_F | 7 | 6.90 | 0.00 | 4.08 | 38.46 | 0.00 | 11.84^**^ | 2.16 | 10.75^**^ | 9.68^**^ | 10p11.2 |
| HS3ST2_P546_F | 12 | 3.45 | 25.00 | 12.24 | 46.15 | 87.50 | 11.79^**^ | 5.24* | 1.57 | 6.55* | 16p12 |
| PDGFRB_P343_F | 9 | 3.45 | 10.00 | 6.12 | 46.15 | 62.50 | 11.72^**^ | 0.87 | 5.61* | 10.85^ϒϒ^ | 5q31-q32 |
| CREB1_P819_F | 5 | 0.00 | 5.00 | 2.04 | 30.77 | 12.50 | 10.77^ϒ^ | 1.82 | 4.08^#^ | 8.95^**^ | 2q34 |
| RARB_P60_F | 9 | 13.79 | 0.00 | 8.16 | 38.46 | 16.67 | 10.77^ϒ^ | 4.44^#^ | 10.75^**^ | 6.33* | 3p24 |
| CDH13_P88_F | 21 | 13.79 | 50.00 | 28.57 | 53.85 | 91.67 | 10.44^ϒ^ | 7.64^ϒ^ | 0.05 | 2.81 | 16q24.2-q24.3 |
| GSTM2_P109_R | 3 | 0.00 | 0.00 | 0.00 | 23.08 | 87.50 | 9.98^ϒ^ | 0.00 | 6.06* | 9.98^**^ | 1p13.3 |
| WT1_P853_F | 6 | 0.00 | 25.00 | 10.20 | 7.69 | 75.00 | 9.88^ϒ^ | 9.80^**^ | 1.75 | 0.08 | 11p13 |
| ICA1_P72_R | 4 | 0.00 | 20.00 | 8.16 | 0.00 | 75.00 | 9.65^ϒ^ | 7.69^ϒ^ | 4.36^#^ | 1.95 | 7p22 |
| MYOD1_E156_F | 13 | 10.34 | 15.00 | 12.24 | 53.85 | 95.83 | 9.53^ϒ^ | 0.24 | 5.63* | 9.30^**^ | 11p15.4 |
| TAL1_P594_F | 20 | 13.79 | 45.00 | 26.53 | 53.85 | 95.83 | 9.23^ϒ^ | 5.90* | 0.25 | 3.33 | 1p32 |
| TIAM1_P188_R | 8 | 6.90 | 30.00 | 16.33 | 0.00 | 37.50 | 8.69* | 4.62^#^ | 6.86^ϒ^ | 4.07^#^ | 21q22.11 |
| TGFBI_P173_F | 6 | 0.00 | 15.00 | 6.12 | 23.08 | 8.33 | 8.47* | 5.66* | 0.34 | 2.81 | 5q31 |
| DSC2_E90_F | 6 | 0.00 | 15.00 | 6.12 | 23.08 | 62.50 | 8.47* | 5.66* | 0.34 | 2.81 | 18q12.1 |
| TAL1_E122_F | 9 | 3.45 | 15.00 | 8.16 | 38.46 | 91.67 | 8.43* | 2.10 | 2.32 | 6.33* | 1p32 |
| HGF_E102_R | 9 | 3.45 | 15.00 | 8.16 | 38.46 | 91.67 | 8.43* | 2.10 | 2.32 | 6.33* | 7q21.1 |
| DCC_P177_F | 6 | 3.45 | 25.00 | 12.24 | 0.00 | 66.67 | 8.23* | 5.24* | 5.58* | 2.99 | 18q21.3 |
| HS3ST2_E145_R | 21 | 17.24 | 55.00 | 32.65 | 38.46 | 95.83 | 7.87* | 7.72^ϒ^ | 0.87 | 0.15 | 16p12 |
| SMARCA3_P17_R | 8 | 6.90 | 5.00 | 6.12 | 38.46 | 4.17 | 7.86* | 0.08 | 6.03* | 7.79^ϒ^ | 3q24 |
| TNFRSF10C_E109_F | 23 | 24.14 | 35.00 | 28.57 | 69.23 | 95.83 | 7.77* | 0.68 | 3.77 | 7.10^ϒ^ | 8p22-p21 |
| HOXC6_P456_R | 11 | 10.34 | 10.00 | 10.20 | 46.15 | 50.00 | 7.73* | 0.00 | 5.61* | 7.72^ϒ^ | 12q13.3 |
| TUSC3_E29_R | 5 | 0.00 | 20.00 | 8.16 | 7.69 | 8.33 | 7.70* | 7.69^ϒ^ | 1.00 | 0.00 | 8p22 |
| PTPN6_E171_R | 8 | 24.14 | 5.00 | 16.33 | 0.00 | 16.67 | 7.69* | 3.62 | 1.02 | 4.07^#^ | 12p13 |
| GNMT_P197_F | 4 | 0.00 | 5.00 | 2.04 | 23.08 | 79.17 | 7.68* | 1.82 | 2.39 | 5.85* | 6p12 |
| CDH1_P45_F | 14 | 27.59 | 30.00 | 28.57 | 0.00 | 45.83 | 7.64* | 0.03 | 6.86^ϒ^ | 7.61^ϒ^ | 16q22.1 |
| PITX2_E24_R | 26 | 24.14 | 60.00 | 38.78 | 53.85 | 66.67 | 7.41* | 6.46* | 0.12 | 0.95 | 4q25-q27 |
| INHA_P1189_F | 3 | 0.00 | 15.00 | 6.12 | 0.00 | 12.50 | 7.12^#^ | 5.66* | 3.20 | 1.45 | 2q33-q36 |
| MAP2K6_P297_R | 3 | 0.00 | 15.00 | 6.12 | 0.00 | 41.67 | 7.12^#^ | 5.66* | 3.20 | 1.45 | 17q24.3 |
| PTGS2_P308_F | 12 | 20.69 | 30.00 | 24.49 | 0.00 | 37.50 | 6.92^#^ | 0.55 | 6.86^ϒ^ | 6.37* | 1q25.2-q25.3 |
| GABRB3_P92_F | 26 | 48.28 | 20.00 | 36.73 | 61.54 | 58.33 | 6.82^#^ | 4.25^#^ | 5.92* | 2.57 | 15q11.2-q12 |
| ZP3_E90_F | 2 | 0.00 | 0.00 | 0.00 | 15.38 | 41.67 | 6.51^#^ | 0.00 | 3.93^#^ | 6.51* | 7q11.23 |
| CDKN2B_seq_50_S294_F | 2 | 0.00 | 0.00 | 0.00 | 15.38 | 16.67 | 6.51^#^ | 0.00 | 3.93^#^ | 6.51* | 9p21 |
| MLH3_P25_F | 2 | 0.00 | 0.00 | 0.00 | 15.38 | 0.00 | 6.51^#^ | 0.00 | 3.93^#^ | 6.51* | 14q24.3 |
| CEBPA_P1163_R | 2 | 0.00 | 0.00 | 0.00 | 15.38 | 83.33 | 6.51^#^ | 0.00 | 3.93^#^ | 6.51* | 19q13.1 |
| DAB2IP_E18_R | 4 | 13.79 | 0.00 | 8.16 | 0.00 | 12.50 | 6.39^#^ | 4.44^#^ | 0.00 | 1.95 | 9q33.1-q33.3 |
| TGFB2_E226_R | 6 | 10.34 | 0.00 | 6.12 | 23.08 | 45.83 | 6.09^#^ | 3.28 | 6.06* | 2.81 | 1q41 |
| IRF7_P277_R | 7 | 6.90 | 5.00 | 6.12 | 30.77 | 25.00 | 5.17 | 0.08 | 4.08^#^ | 5.10* | 11p15.5 |
| MME_P388_F | 7 | 6.90 | 5.00 | 6.12 | 30.77 | 75.00 | 5.17 | 0.08 | 4.08^#^ | 5.10* | 3q25.1-q25.2 |
| SCGB3A1_E55_R | 14 | 10.34 | 35.00 | 20.41 | 30.77 | 83.33 | 5.00 | 4.40^#^ | 0.06 | 0.60 | 5q35-qter |
| DLK1_E227_R | 14 | 10.34 | 35.00 | 20.41 | 30.77 | 91.67 | 5.00 | 4.40^#^ | 0.06 | 0.60 | 14q32 |
| WNT1_P79_R | 11 | 6.90 | 30.00 | 16.33 | 23.08 | 83.33 | 4.93 | 4.62^#^ | 0.19 | 0.31 | 12q13 |
| IRAK3_E130_F | 13 | 10.34 | 35.00 | 20.41 | 23.08 | 91.67 | 4.44 | 4.40^#^ | 0.54 | 0.04 | 12q14.3 |

The log-likelihood G statistic was used to calculate p-values. This is an alternative to a chi-square test that is more accurate when dealing with low expected values. **All G-value** = VHL vs Sporadic VHLwt ccRCC vs Papillary RCC (2df). **Clear G value** = VHL vs sporadic VHLwt ccRCC (1df). **Spor G value** = Sporadic VHLwt ccRCC vs papillary RCC (1df). **Pap v cc G value** = All ccRCC (VHL and VHLwt sporadic) vs papillary RCC (1df (df = degrees of freedom).

Key for p-values: ^#^ =P<0.05, *=P<0.025, ^ϒ^ =P<0.01, **=P<0.005 and ^ϒϒ^ =P<0.001).

Cyto_Map = cytogenetic map position. TargetID describes the particular CpG represented on the Illumina array: e.g. E116 = CpG at position 116 downstream in the first exon; P585 = CpG at position 585 upstream in the promoter. F and R (forward and reverse) refer to which strand of the DNA is targeted.
